# Improvements in interoceptive sensibility, pain and functional clinical outcome measures after participation in an interdisciplinary pain management program

**DOI:** 10.1002/pmrj.70110

**Published:** 2026-03-16

**Authors:** Stephanie Voss, Shannon La Penna, Alison Yum, Neha P. Gothe, Christine M. Gagnon

**Affiliations:** ^1^ Health and Kinesiology Department University of Illinois Urbana‐Champaign Urbana Illinois USA; ^2^ Pain Management Center Shirley Ryan AbilityLab Chicago Illinois USA; ^3^ Bouvé College of Health Sciences Northeastern University Boston Massachusetts USA; ^4^ Physical Medicine and Rehabilitation Department Northwestern University Chicago Illinois USA

## Abstract

**Background:**

Altered interoception, the sense of signals from inside the body, is linked to chronic pain. No studies to date have examined whether interoceptive sensibility, one's self‐reported beliefs about the body and tendencies to be internally focused, changes following participation in an interdisciplinary pain management program (IPMP).

**Objective:**

The primary aim was to determine whether interoceptive sensibility changes following IPMP participation. The second aim was to explore whether change in interoceptive sensibility could explain improvement in functional outcomes.

**Methods:**

An observational cohort study was conducted at an outpatient pain management center housed within an urban academic rehabilitation center. A heterogeneous sample of  53 (mean age = 50.3, 81.1% female) patients with high‐impact chronic pain completed study measures in addition to receiving interdisciplinary care with physical therapy, occupational therapy, and psychology. Measures included the Multidimensional Assessment of Interoceptive Awareness (MAIA‐2), Patient‐Reported Outcomes Measurement Information System Pain Intensity and Interference short forms, Canadian Occupational Performance Measure (COPM), and the Patient Specific Functional Scale. Paired *t*‐tests examined change in outcomes from program start to program completion. Hierarchical linear regressions tested the unique contribution of change in interoceptive sensibility on functional outcomes.

**Results:**

There was a significant increase in interoceptive sensibility and improvement on all pain and functional outcomes (*p*'s < .001, absolute *d*'s = 0.70–1.55). Six of the eight MAIA‐2 subscales increased significantly (*p*'s < .001, *d*'s = 0.42–0.83), with the greatest increase in the Self‐Regulation and Not Worrying subscales. An increase in overall interoceptive sensibility contributed an additional 6.3% (*β* = 0.267, *p* < .05) and 15.8% (*β* = 0.424, *p ≤* .001) to the variance in improved COPM‐Performance and COPM‐Satisfaction scores after program completion, respectively.

**Discussion:**

This study provides emerging evidence that interoceptive sensibility improves following IPMP participation and is associated with improved functional engagement in high‐impact chronic pain.

**Conclusion:**

IPMPs may be uniquely suited to improving interoceptive self‐regulation, which could be an important treatment target in chronic pain. More research is needed to examine the role of interoceptive sensibility in different pain conditions as well as the impact of cultural, demographic, and psychosocial differences on interoceptive sensibility in chronic pain.

## INTRODUCTION

In the United States, chronic pain affects ∼20% of adults. An estimated 10% of these individuals experience high‐impact chronic pain, resulting in significant functional disability and difficulty performing self‐care, leisure, and productivity‐related tasks.^1^, ^2^, ^3^, ^4^ Thus, there is an urgent need to identify patient‐specific treatment targets to improve pain and function.

Interdisciplinary pain management programs (IPMPs) are the preferred treatment approach for high‐impact chronic pain.^4^, ^5^, ^6^ Rooted in biopsychosocial models, IPMPs are patient centered and well suited to address the complex impact of chronic pain on physical health, psychological well‐being, and daily functioning. Interdisciplinary teams typically include nonopioid pharmacological management, physical therapy (PT), occupational therapy (OT), psychotherapy, and vocational rehabilitation. Some programs also integrate complementary approaches like mindfulness and tai chi.^4^, ^5^, ^6^, ^7^ In a recent review of longitudinal outcomes from 72 patient cohorts, IPMPs improved a variety of functional outcomes including pain, physical function, depression, anxiety, and social functioning. Importantly, these improvements were largely maintained longterm.^7^ Identifying patient factors associated with improved functional engagement after IPMP participation may help identify valuable therapeutic targets to improve chronic pain.

Accumulating evidence suggests that altered interoception, the sense of internal bodily signals, is linked to chronic pain.^8^, ^9^ Interoceptive sensibility is one dimension of interoception and refers to one's self‐reported beliefs about the body and one's tendencies to be internally focused.^10^, ^11^ Assessing differences in interoceptive sensibility could provide clinicians with valuable insight into a patient's relationship with their body to inform treatment approaches. For example, patients with decreased trust in body sensations may benefit from pain science education and a graded approach to exercise to understand the protective role of pain and relearn how to identify safe movement patterns. However, although enhanced interoceptive sensibility is increasingly tied to improved pain,^9^, ^12^ no studies to date have examined whether interoceptive sensibility improves after IPMP participation or whether interoceptive sensibility contributes to improved function.

Therefore, this study examined the relationship between interoceptive sensibility and functional outcomes in a high‐impact chronic pain population after IPMP participation. The specific aims were (1) to determine whether interoceptive sensibility changes following IPMP participation and (2) to explore whether change in interoceptive sensibility could explain improvement in functional outcomes.

## METHODS

### Procedures

Participants were recruited from an outpatient pain management center housed within an urban academic rehabilitation center on a rolling basis over a 13‐month period. Potential participants were prescreened for study eligibility. Eligible patients were invited to participate in the study in addition to receiving their regularly scheduled care. Inclusion criteria sought to broadly represent the patient population typically seen in this setting and included enrollment in the IPMP, age ≥ 18 years, and English speaking. Exclusion criteria included enrollment in individual services only (received no group therapies), diagnosed with a functional movement/neurological disorder, diagnosed with postural orthostatic tachycardia syndrome, treatment associated with a worker's compensation claim, and inability to provide informed consent. Participation was voluntary and written informed consent was obtained from all participants. Participants were compensated up to $10 ($5 at each time point) for their time. This study was approved by the Northwestern University Institutional Review Board (IRB# STU00217991) and conducted in compliance with the Helsinki accords and HIPAA law.

### Interdisciplinary pain management program

The IPMP regularly treats a diverse group of chronic pain syndromes and IPMP participation was part of all participants' standard of care. Coordinated services were provided by multiple providers in the same location that communicated daily, progressed toward similar goals, and engaged in shared decision‐making with patients. Patients received a mixture of individual and group therapies from PT, OT, and psychology, regular check‐ins with pain physicians, and vocational counseling as needed. IPMP outcome measures focus on function rather than pain, and measure factors related to quality of life, physical and psychosocial function, and perceived functional engagement in activities of daily living.

All disciplines regularly address pain education, confidence, and fear of movement throughout program participation and therapeutic services are tailored to the individual. In PT, approaches include graded return to exercise, aerobic exercise, strengthening, stretching, and balance training. In OT, approaches include graded motor imagery, pacing, sensory strategies, posture/body mechanics, leisure participation, and routine development. In psychology, approaches include cognitive behavioral therapy (CBT), acceptance and commitment therapy, mindfulness‐based stress reduction, and biofeedback. Examples of group sessions include strength and conditioning in PT, mindfulness and relaxation in psychology, and functional mechanics and routine development groups in OT. Complementary approaches are also integrated and delivered in a group format, including yoga, tai chi, Feldenkrais, and mindfulness‐based relaxation.

Individual IPMP participation varied between patients and was representative of the patient population in this setting. Plans of care are determined on an individual basis depending on presentation complexity, insurance limitations, and therapeutic needs. Programs thus varied in frequency and duration (1–5 days per week for 4–6 weeks) ranging from 6 to 35 hours per week. Patients in the full or modified‐full program received 4–5 days of therapy for 4 weeks; partial program patients received 1–3 days of therapy for 6 weeks. All enrolled participants received group therapy from each discipline.

### Measures

Participants routinely complete several questionnaires during initial evaluation and discharge assessments. As part of this study, participants were also asked to complete the Multidimensional Assessment of Interoceptive Awareness, Version 2 (MAIA‐2) and the Patient‐Reported Outcomes Measurement Information System (PROMIS) measures of pain intensity and pain interference. Remaining study measures were collected from the electronic medical record and included demographic information (age, gender, race or ethnicity), program type (days/week, total weeks), pain diagnosis, and functional outcome measures administered by PT and OT. The measures are described briefly below; scoring and psychometrics are further detailed in the Supplementary Material.

#### Multidimensional Assessment of Interoceptive Awareness, Version 2

The MAIA‐2 is a 37‐item questionnaire measuring eight domains of interoceptive sensibility, including Noticing, Not Distracting, Not Worrying, Attention Regulation, Emotional Awareness, Self‐Regulation, Body Listing, and Trusting. The original MAIA was developed to distinguish between separate aspects of interoceptive sensibility, such as tendencies to be internally focused or beliefs about pain.^13^, ^14^ Although designed for subscale interpretation, this study also calculated a mean MAIA‐2 score to represent overall interoceptive sensibility, as in previous studies.^15^, ^16^, ^17^ Higher scores indicate greater interoceptive sensibility.

#### Patient Reported Outcomes Measurement Information System – Pain Intensity and Pain Interference forms

The PROMIS Pain Intensity and Pain Interference forms are part of the National Institutes of Health toolbox of patient‐reported outcomes.^18^ The Pain Intensity measure is a three‐item questionnaire, providing a quantitative assessment of pain intensity over the past 7 days at its worst, average, and “right now.” The Pain Interference Short Form 8a is an eight‐item questionnaire providing a global rating of how much pain interferes with daily life, including activities of daily living, social activities, and leisure. Individual scores correspond to T‐Scores (mean = 50, SD = 10), with higher scores indicating greater levels of pain intensity and interference.

#### Canadian Occupational Performance Measure

The Canadian Occupational Performance Measure (COPM) is a measure of functional engagement, designed to assess an individual's perceived level of performance and satisfaction with up to five valued occupations in the context of injury or illness.^19^ The COPM enables patients to participate in the therapy process by collaborating with OTs to identify their most important occupational performance problems. Ratings are summed and divided by the number of responses to yield a total score. Higher scores indicate greater functional engagement with patient‐valued goals.

#### Patient‐Specific Functional Scale

The Patient‐Specific Functional Scale (PSFS) measures a patient's subjective report of functional impairments due to an injury or illness with up to three patient‐specific functional goals.^20^ Ratings are summed and divided by the number of responses to yield a total score. Lower scores indicate greater functional limitation.

### Sample size and power

An a priori sample size calculation using G*Power (version 3.1.9.7) was performed to determine the samples needed to detect change in the primary overall interoceptive sensibility variable. For a two‐tailed paired sample *t*‐test with overall MAIA‐2 as the dependent variable, we selected an effect size of *d* = 0.40,^16^, ^21^, ^22^ alpha = 0.05, and target power = 0.80. The calculation resulted in a recommended sample size of *N* = 52.

### Statistics

The data were examined for outliers (*Z* = ±3.29) and normality (Shapiro–Wilk) prior to conducting statistical analyses. No outliers were detected. Age, pain intensity, and COPM‐Satisfaction were not normally distributed. Age had a bimodal distribution. There were some missing data (patient specific functional scale [PSFS], 13.2% and < 2% all other variables); analyses were conducted by excluding cases on an analysis‐by‐analysis basis in order to retain as much of the data as possible. The planned statistical analyses are robust to non‐normality given the relatively large sample sizes on all measures. Assumptions were met for all statistical analyses.

Paired samples *t*‐tests were used to compare changes in outcomes from baseline to program completion. Age was not used as a covariate due to the large impact (reduction) on the observed power with its inclusion. Furthermore, we performed a median split on age and compared the two groups on the change scores of all outcomes and there were no significant differences between younger and older participants. A standardized measure of effect size for paired samples, Cohen's *d*, was used to indicate the magnitude of the observed differences in pre‐ to posttreatment scores.^23^ Effect sizes of ±0.2, 0.5, and 0.8, indicate small, medium, and large effects, respectively.^24^ Due to the large number of comparisons, the statistical significance level for the primary MAIA‐2 outcome was set to *p* < .05, with the remaining *t*‐tests set to a conservative *p* < .001. Exploratory hierarchical linear regressions were conducted to test for the unique contribution of the MAIA‐2 total score. The significance level was set at *p* < .05. Pearson's partial correlations, controlled for age, were used to explore associations between the MAIA‐2 scores and pain (intensity and interference) and between the MAIA‐2 subscales and clinical outcomes. The statistical significance level was set at *p* < .05 for these exploratory correlation analyses.

## RESULTS

### Participant characteristics

A total of 229 patients were prescreened for eligibility, of which 82 met one or more exclusion criteria described previously (eg, worker's compensation, non‐English speaking, postural orthostatic tachycardia syndrome diagnosis etc.). Of the 147 patients eligible to contact, 63 enrolled and 53 completed the program (Figure 1). Demographics and pain diagnostic criteria are described in Tables 1 and 2. There were no significant baseline differences between participants who did and did not complete the program, except that noncompleters reported a greater ability to control attention to bodily sensations (*p* = .04) and a lower tendency to worry about pain (*p* = .01), which theoretically could contribute to program dropout due to lower needs (Table S2). There were no significant differences in baseline measures between patients in the full (4‐week) versus partial (6‐week) programs (all *p*'s > .05). The remaining results report outcomes for program completers only and were combined for program type.

**FIGURE 1:**
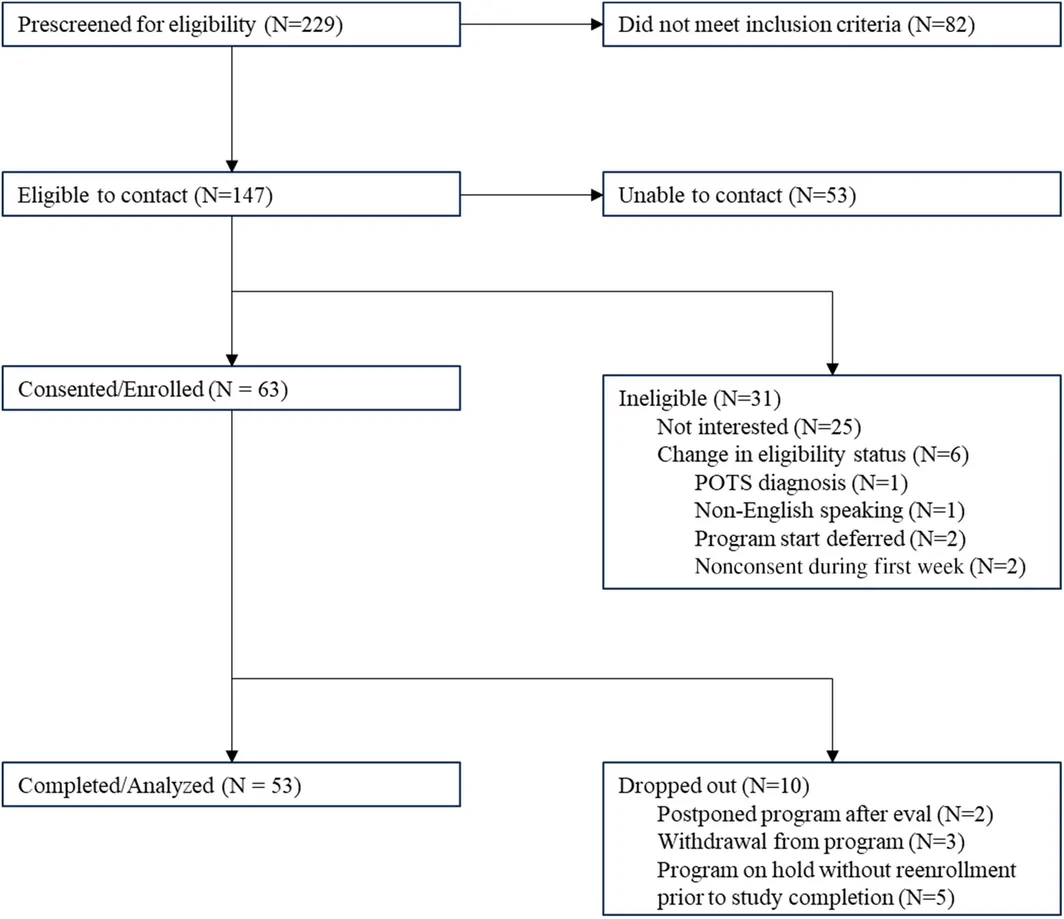
Participant flow diagram. POTS, postural orthostatic tachycardia syndrome.

**TABLE 1:** Participant characteristics.

	All participants	Completers	Noncompleters	*t*‐test/*χ* ^2^
*N*	63	53	10	
Age, y, mean (SD)	50.9 (17.5)	50.3 (17.3)	55.2 (17.9)	*p* = .40
Range	23–81	23–81	26–74	
Female gender, *N* (%)	49 (77.8%)	43 (81.1%)	6 (60%)	*p* = .14
Race, *N* (%)				
Black/African American	9 (14.3%)	5 (9.4%)	4 (40%)	
White	41 (65.1%)	37 (69.8%)	4 (40%)	
Asian Indian	3 (4.8%)	3 (5.7%)	0	*p* = .12
Other	5 (7.9%)	4 (7.5%)	1 (10%)	
Declined to answer	5 (7.9%)	4 (7.5%)	1 (10%)	
Hispanic/Latino, *N* (%)				
Yes	1 (1.6%)	1 (1.9%)	0	
No	56 (88.9%)	47 (88.7%)	9 (90%)	*p* = .91
Declined to answer	6 (9.5%)	5 (9.4%)	1 (10%)	
Program duration, *N* (%)				
4‐week program	23 (36.5%)	20 (37.7%)	3 (30%)	*p* = .64
6‐week program	40 (63.5%)	33 (62.3%)	7 (70%)	

**TABLE 2:** Pain characteristics.

	All participants	Completers	Noncompleters
Primary pain symptom, *N* (%)			
One pain symptom	7 (11.1%)	7 (13.2%)	0
Multiple pain symptom	56 (88.9%)	46 (86.8%)	10 (100%)
Primary pain region/type, *N* (%)			
Cervical	8 (12.7%)	7 (13.2%)	1 (10%)
Thoracic	2 (3.2%)	1 (1.9%)	1 (10%)
Lumbar	20 (31.7%)	18 (34%)	2 (20%)
Fibromyalgia	9 (14.3%)	8 (15.1%)	1 (10%)
Generalized/multiregion	6 (9.5%)	4 (7.5%)	2 (20%)
Lower extremity	4 (6.3%)	3 (5.7%)	1 (10%)
Upper extremity	2 (3.2%)	2 (3.8%)	0
Neuralgia	2 (3.2%)	1 (1.9%)	1 (10%)
Radiculopathy	1 (1.6%)	0	1 (10%)
Complex regional pain syndrome	2 (3.2)	2 (3.8%)	0
Headache/migraine	2 (3.2)	2 (3.8%)	0
Other	5 (7.9%)	5 (9.4%)	0

Participants varied widely in age, were predominantly female, White, and non‐Hispanic/Latino (Table 1). Most were enrolled in a 6‐week partial program of which *N* = 4, 12, 11 and 6 completed 1 full day, 2 half days, 2 full days, and 3 full days, respectively. Participants enrolled in a 4‐week program included *N* = 7 and 13 completing 4 and 5 full days, respectively. Most participants reported more than one pain symptom, and the most common primary pain types were lumbar pain, fibromyalgia, and cervical pain (Table 2).

### Baseline measures

Participants reported above average pain intensity and interference, low COPM‐Performance and COPM‐Satisfaction, high perceived PSFS limitation, and an overall mean interoceptive sensibility of 2.61 (0.61) (Table 3). Interoceptive sensibility differed between subscales and was lowest for Not Distracting and highest for Noticing and Emotional Awareness (Table S2).

Significant partial correlations between baseline pain and functional measures were in the expected direction (*p*'s < .05) and higher overall interoceptive sensibility was weakly associated with greater perceived COPM‐Performance (*r* = 0.282, *p* = .043) and COPM‐Satisfaction (*r* = 0.297, *p* = .032) (Table S3). Associations between baseline MAIA‐2 subscales and pain and functional measures varied widely. There were a few significant and weak partial correlations (*r*'s = 0.274–0.429; *p*'s < .05) with an initial pattern linking greater pain intensity and interference to higher Noticing and Body Listening scores (Table S4).

### Changes after IPMP completion

Paired samples *t*‐tests revealed a significant increase in overall interoceptive sensibility, reduced pain intensity and interference, and improvement on all functional outcome measures (all *p*'s < .001, absolute *d*'s = 0.70–1.55) (Table 3). These improvements met clinically meaningful criteria for all measures, except for the MAIA‐2 total score change, as the criteria for the MAIA‐2 have yet to be established. Both older and younger participants demonstrated significantly increased MAIA‐2 but the change in interoceptive sensibility did not differ by age (younger group mean change = 0.65 [0.45], older group mean change = 0.45 [0.37]; *p* > .05) and were thus combined. Mean improvements for pain and functional outcomes were all within previously reported minimal important difference/minimal clinically important difference ranges.^19^, ^25^, ^26^ There was a statistically significant increase on six of the eight MAIA‐2 subscales (*p*'s < .001, *d*'s = 0.42–0.83) (Table 4). The increase in the Noticing (*p =* .001, *d* = 0.37) and Not Distracting (*p* = .002, *d* = 0.50) scales did not survive correction for multiple comparisons with a conservative *p* < .001. The magnitude in change following IPMP participation was largest for Self‐Regulation (mean change = 0.79, *d* = 0.83) and Not Worrying (mean change = 0.68, *d* = 0.74) and smallest for Noticing (mean change = 0.35, *d* = 0.37) and Not Distracting (mean change = 0.40, *d* = 0.50).

**TABLE 3:** Change in overall interoceptive sensibility, pain, and functional measures after IPMP completion.

	Initial (mean, SD)	Discharge (mean, SD)	ΔMean (SD)	MID/MCID	T (df)	*d*
MAIA‐2, overall	2.56 (0.60)	3.12 (0.53)	0.55 (0.42)	n/a	9.39 (51)	0.97
PROMIS pain						
Intensity	56.50 (4.85)	52.91 (5.28)	−3.57 (5.05)	> −2–6^26^	5.10 (51)	−0.70
Interference	66.60 (5.46)	61.89 (6.61)	−4.50 (5.91)	> −2–6^26^	5.49 (51)	−0.74
Functional outcomes						
COPM‐P	3.51 (1.00)	5.51 (1.94)	1.98 (1.67)	> +2^19^	8.56 (51)	1.18
COPM‐S	2.60 (1.31)	5.62 (2.32)	3.01 (2.30)	> +2^19^	9.47 (51)	1.55
PSFS	3.13 (1.20)	5.51 (1.95)	2.34 (1.75)	> +2^20^	9.10 (45)	1.38

*Note:* Results of paired sample *t*‐tests, all *p*'s < .001. PROMIS MID values vary by population; PROMIS developers recommend 2–6 T‐score points as reasonable for most contexts.^26^

Abbreviations: Δ, change; COPM‐P/S, Canadian Occupational Performance Measure‐Performance/Satisfaction; *d*, Cohen's *d*; IPMP, interdisciplinary pain management program; MAIA‐2, Multidimensional Assessment of Interoceptive Awareness, Version 2; MID/MCID, minimal important difference/minimal clinically important difference; PSFS, Patient‐Specific Functional Scale.

**TABLE 4:** Change in MAIA‐2 subscales after IPMP completion.

MAIA‐2 subscale	Initial (mean, SD)	Discharge (mean, SD)	ΔMean (SD)	*T* (df)	*d*
Noticing	3.36 (1.02)	3.71 (0.84)	0.35 (0.74)	3.44 (51)	0.37
Not distracting	1.75 (0.90)	2.15 (0.68)	0.40 (0.90)	3.23 (51)	0.50
Not worrying	2.29 (0.96)	2.97 (0.89)	0.68 (0.79)	6.19 (51)	0.74
Attention regulation	2.69 (1.00)	3.28 (0.74)	0.59 (0.77)	5.53 (51)	0.64
Emotional awareness	3.27 (0.98)	3.78 (0.74)	0.51 (0.71)	5.21 (51)	0.56
Self‐regulation	2.43 (1.00)	3.22 (0.89)	0.79 (0.86)	6.62 (51)	0.83
Body listening	2.53 (1.22)	3.02 (1.06)	0.49 (0.88)	4.01 (51)	0.42
Trusting	2.37 (1.48)	3.00 (1.27)	0.64 (1.10)	4.18 (51)	0.45

*Note*: Results of paired sample *t*‐tests. All *p*'s < .001 except Noticing (*p* = .001) and Not Distracting (*p* = .002).

Abbreviations: Δ, change; *d*, Cohen's *d*; IPMP, Interdisciplinary Pain Management Program; M, mean; MAIA‐2, Multidimensional Assessment of Interoceptive Awareness, version 2.

### Regression results

A series of hierarchical regression analyses were conducted to explore if changes in overall interoceptive sensibility on the MAIA‐2 contributed to changes in functional outcomes beyond those accounted for by demographics and pain interference. The change scores for the functional program outcomes (COPM‐Performance, COPM‐Satisfaction, PSFS) served as the criterion variables while age, gender, pain interference change scores, and overall MAIA‐2 change scores served as the predictor variables. The overall MAIA‐2 change score was selected as the primary predictor variable. Pain intensity was not included in any of the models due to multicollinearity with pain interference. In each of the regression models, variables were entered in a stepwise approach with demographics entered first, followed by change in pain interference, and then change in MAIA‐2 (*p* < .05 to enter, *p* > .10 to remove).

The results of the regression analyses show that the average unique contribution (mean change in R^2^) for the three functional outcome variables was 2% for the demographics, 34.5% for pain interference, and the MAIA‐2 score contributed an additional 7.4% to the variance (total variance = 43.9%) (Table 5). The demographic variables (age and gender) were nonsignificant in all three regression models. Pain interference contributed significantly to all three models (change *R* ^2^ = 37.4% for COPM‐Performance. 39.6% COPM‐Satisfaction. and 26.5% for PSFS). Change in MAIA‐2 contributed significantly to the variance in two of the three models COPM‐Performance (change *R* ^2^ = 6.3%), and COPM‐Satisfaction (change *R* ^2^ = 15.8%).

**TABLE 5:** Hierarchical regressions examining prediction of improvements in program outcomes.

Outcome	Predictors	*R*	*R* ^2^	Change *R* ^2^	*β*(final)
COPM performance					
Model	1. Demographics	0.133	0.018	0.018	
	Age				−0.029
	Gender				−0.019
	2. Pain interference	0.626	0.392	0.374	−0.547***
	3. MAIA‐2	0.674	0.454	0.063	0.267*
COPM satisfaction					
Model	1. Demographics	0.170	0.029	0.029	
	Age				−0.031
	Gender				0.018
	2. Pain interference	0.652	0.425	0.396	−0.525***
	3. MAIA‐2	0.763	0.583	0.158	0.424***
PSFS total score					
Model	1. Demographics	0.113	0.013	0.013	
	Age				−0.020
	Gender				0.061
	2. Pain interference	0.527	0.278	0.265	−0.512***
	3. MAIA‐2	0.528	0.279	0.001	0.031

Abbreviations: COPM‐P/S, Canadian Occupational Performance Measure‐Performance/Satisfaction; MAIA‐2, Multidimensional Assessment of Interoceptive Awareness, version 2; PSFS, Patient‐Specific Functional Scale.

* *p* < .05;

*** *p* ≤ .001.

## DISCUSSION

The results of this study identified a significant increase in interoceptive sensibility and improvement in pain and functional outcomes after IPMP participation. Functional improvements were statistically and clinically meaningful, lending support to previous findings of the benefits of IPMP for chronic pain management.^7^ An increase in overall interoceptive sensibility contributed an additional 6.3% and 15.8% to explaining the variance of improved COPM‐Performance and COPM‐Satisfaction scores after program completion, respectively. These findings suggest that an increase in overall interoceptive sensibility may be associated with improved functional engagement after IPMP participation.

### Aim 1

Interoceptive sensibility was broadly responsive to interdisciplinary care, as indicated by an increase in overall interoceptive sensibility and six of the eight MAIA‐2 subscales, all with medium‐to‐large effect sizes. The remaining two subscales (Noticing and Not Distracting) also increased substantially but did not survive correction for multiple comparisons with a conservative *p* < .001. Although self‐compassion training, tai chi, qigong, Feldenkrais, and mindfulness‐based approaches have also improved interoceptive sensibility in chronic pain populations, these gains are often limited to a few subscales, such as Self‐Regulation and Trusting.^15^, ^16^, ^17^, ^22^, ^27^, ^28^ Coordinated care from several providers is an essential component of interdisciplinary pain management and may have contributed to increases across the behavioral, attentional, and emotional domains of the MAIA‐2. As the preferred treatment approach for high‐impact chronic pain, these data may serve as a useful benchmark against which future studies can examine whether certain pain populations or deficits in interoceptive sensibility would benefit from targeted intervention approaches.

The MAIA‐2 subscale most responsive to change was Self‐Regulation, suggesting that IPMPs are particularly well equipped to train the active and intentional use of the body to regulate distress. We highlight the importance of interoceptive self‐regulation as a component of interoceptive sensibility due to the high degree of attention to the body—and potentially symptoms—involved in body‐oriented treatment approaches. As pain catastrophizing is one of the most consistent constructs associated with chronic pain,^29^ body‐oriented approaches that enhance interoceptive attention could increase distress if delivered in the absence of concurrent self‐regulatory training.^11^ The clinical implications of these findings are discussed further later.

### Aim 2

Increased interoceptive sensibility made a significant contribution to explaining the variance in improved COPM scores while accounting for the variance explained by demographics and reductions in pain interference. There was a greater contribution to the model for COPM‐Satisfaction than COPM‐Performance but no significant contribution to the PSFS. Given the similarities between the COPM and PSFS, these findings were unexpected.

A few differences between the measures may explain why interoceptive sensibility was related to the COPM but not the PSFS. First, this study used the three‐item version of the PSFS whereas the COPM has five items; fewer items may result in lower internal consistency. However, both the COPM‐Performance and the PSFS had similarly low Cronbach's alphas at baseline (*α*'s = 0.502 and 0.525, respectively) and both alphas improved at posttesting (*α*'s = 0.859 and 0.811, respectively). These data are consistent with previous findings reporting questionable construct validity of the three‐item PSFS in a heterogeneous sample of high‐impact chronic pain.^30^ As both the COPM and PSFS ask patients to identify patient‐specific functional impairments and goals, the levels of physical function could vary widely both within and across individual's responses. For example, a patient may report low ability to run one mile on the PSFS and select the goal of donning shoes independently on the COPM.

Second, changes in interoceptive sensibility may be more related to changes in satisfaction than changes in physical function. Perceived satisfaction, regardless of physical function, could be a more consistent construct in a heterogeneous population. The COPM‐Satisfaction scale did indeed have stronger internal consistency at both pre‐ and posttesting (*α*'s = 0.708 and 0.869, respectively). Furthermore, the COPM is validated against measures of quality of life and psychological functioning and likely captures the impact of chronic pain beyond physical ability.^19^, ^31^, ^32^, ^33^ Our findings thus indicate that interoceptive sensibility contributes more to an individual's perceived satisfaction and quality of life with chronic pain than it contributes to physical function.

### Clinical implications

It is often implied that enhanced interoceptive sensibility is a positive outcome for individuals with chronic pain.^9^, ^12^ Although our findings suggest that increased overall interoceptive sensibility is indeed linked to improved functional engagement, our exploratory analyses suggest that higher interoceptive sensibility may also reflect hypervigilance toward internal sensations. Prior to intervention, greater pain intensity and interference were partially correlated, albeit weakly, with higher Noticing and Body Listening scores. This emerging pattern has substantial therapeutic implications, such that strategies targeting body awareness (eg, postural training, body scanning, breath awareness) could increase distress if patients are unable to attend to sensations without fear or catastrophizing. The combination of hypervigilance toward internal sensations and reduced interoceptive self‐regulation may benefit from treatment approaches that balance noticing uncomfortable sensations with learning how to monitor and interpret pain as information rather than threat.

There are a few strategies integral to the IPMP approach that may be particularly useful for training interoceptive self‐regulation. First, it is worth assessing whether low Self‐Regulation scores simply reflect unfamiliarity with body‐based regulation strategies, like breathwork, or if patients find such strategies bothersome. Patients that cope with pain by disconnecting from their bodies could find interoceptive input overwhelming.^34^ Such patients may benefit from gradual exposure to interoceptive sensations and validation that relaxation techniques, like breathwork or mindfulness, can be challenging. Second, pacing strategies may be particularly useful for targeting interoceptive self‐regulation. Pacing is regularly integrated into PT and OT sessions and requires patients to attend to and monitor pain levels to inform and adjust activity participation. Symptoms are used to help patients understand when pain levels are safe or when pain levels indicate that adjustment is needed. Third, IPMP's integrate interventions like CBT and mindfulness that complement pacing to support the development of interoceptive self‐regulation. Patients with cognitive distortions about pain may benefit from a greater emphasis on CBT. Mindfulness and relaxation approaches that direct attention to interoceptive sensations (eg, diaphragmatic breathing, biofeedback) can teach patients how to engage with bodily sensations adaptively. Future studies would benefit from examining whether single modality interventions like CBT are similarly or more effective than IPMPs at training interoceptive self‐regulation in chronic pain.

### Limitations

There are several limitations to this study. The observational study design limits evaluation of the effectiveness of the IPMP on interoceptive sensibility against other treatment approaches or nontreatment of chronic pain. The hierarchical regression and correlation analyses were exploratory and thus not corrected for multiple comparisons, potentially increasing the risk of Type 1 errors. These analyses would benefit from replication in larger samples to confirm the results. The heterogeneous sample of high‐impact chronic pain varied in age, diagnostic subgroups, and program type. Although age was included as a covariate in the exploratory correlation and regression analyses, the inclusion of age as covariate for the paired samples *t*‐tests greatly decreased the statistical power. Thus, future studies should further examine the role of age on interoceptive sensibility in chronic pain. Due to scheduling flexibility, there was variability in the types of group therapies received as well as program intensity (eg, full‐time program participants received more intensive care than partial program patients), which may have affected the results. This design was intentional to capture a representative sample of IPMP settings. However, despite significant heterogeneity in age, pain diagnostics, and program enrollment, we were able to combine data for analysis of the full sample. This may be a relative strength of these findings as they are not limited to a specific subpopulation and may be more generalizable to a variety of chronic pain populations, as is typical within IPMPs.

This sample was predominantly White and female and thus lacking demographic diversity. Age, cultural, racial, and gender differences may influence interoceptive sensibility, especially regarding the quality of attention towards and the meaning ascribed to unpleasant sensations. A recent validation study of the Japanese MAIA found a separate factor structure from the original MAIA, and qualitative interviews revealed that concepts like interoceptive self‐regulation served social rather than homeostatic purposes.^35^ Gender differences are also recognized across interoceptive domains. Females typically demonstrate lower interoceptive accuracy but increased attention toward interoceptive sensations; similar patterns appear in chronic pain and autism spectrum disorders.^9^, ^36^, ^37^ One hypothesis suggests that because females experience more periods of discomfort during physical and hormonal change (eg, pregnancy and menopause), they may develop heightened interoceptive attention and become predisposed to noticing and reporting more somatic symptoms.^36^

We also identified higher baseline scores among non‐completers for the Attention Regulation and Not Worrying scales. Individuals with fewer tendencies to worry about pain may have found less benefit from the IPMP and may have been less willing to continue with the time‐intensive program. Furthermore, a greater proportion of Black than White individuals did not complete the program. Although these differences were not statistically significant, it is possible that racial differences could have contributed to baseline differences in interoceptive sensibility between completers and noncompleters.

Lastly, this study did not control for common comorbidities in high‐impact chronic pain. Anxiety, trauma, and neurodevelopmental conditions like autism and attention deficit hyperactivity disorder increasingly demonstrate interoceptive deficits.^34^, ^37^, ^38^ Multisensory sensitivity to interoceptive and environmental stimuli is also linked to chronic pain.^39^, ^40^, ^41^ Sensory processing differences could predispose neurodiverse individuals to greater awareness of, and potential difficulty regulating, interoceptive input. Future large studies would thus benefit from examining how interoceptive sensibility interacts with comorbidities and individual psychosocial factors to influence pain and function.

## CONCLUSION

This study provides emerging evidence that interoceptive sensibility improves following IPMP participation and is associated with improved functional engagement in high‐impact chronic pain. IPMPs may be uniquely suited to improving interoceptive self‐regulation, which could be an important treatment target for chronic pain. Overall interoceptive sensibility and six of the eight MAIA‐2 subscales increased with medium‐to‐large effect sizes and interoceptive sensibility contributed significantly to explaining improved performance and satisfaction with functional engagement. Future research would benefit from follow‐up assessments to examine whether improvements in interoceptive sensibility, pain and function after IPMP treatment are maintained long term. Randomized controlled trials should examine the efficacy of IPMPs on interoceptive sensibility in comparison to single‐modality interventions, like CBT. Studies examining the efficacy of novel interventions may benefit from comparison to IPMPs as the preferred approach for chronic pain treatment. Studies in mixed pain populations would benefit from subgroup analyses to examine whether interoceptive sensibility differs between pain conditions. Individual differences in culture, demographics, psychosocial factors, and comorbidities may also influence interoceptive sensibility and warrant further study. Lastly, no studies to date have examined whether or how much change in interoceptive sensibility is clinically meaningful, which will be essential for understanding how interoceptive sensibility affects pain care.

## FUNDING INFORMATION

This study was funded by the James Brown IV Allied Health Fellowship, Shirley Ryan AbilityLab, Chicago IL. The funding source had no role in the study design, analysis of data, interpretation of results, or writing of the report.

## DISCLOSURE

The authors report no actual or perceived conflicts of interest in the conduct and reporting of this research.

This journal‐based CME activity is designated for 1.0 *AMA PRA Category 1 Credit* ^TM^. Effective January 2024, learners are no longer required to correctly answer a multiple‐choice question to receive CME credit. Completion of an evaluation is required, which can be accessed using this link, https://onlinelearning.aapmr.org/. This activity is FREE to AAPM&R members and available to nonmembers for a nominal fee. CME is available for 3 years after publication date. For assistance with claiming CME for this activity, please contact (847) 737–6000. All financial disclosures and CME information related to this article can be found on the Online Learning Portal (https://onlinelearning.aapmr.org/) prior to accessing the activity.

## Supporting information


**Data S1.** Supporting Information.

## Data Availability

The data that support the findings of this study are available on request from the corresponding author. The data are not publicly available due to privacy or ethical restrictions.

## References

[1] Rikard SM , Strahan AE , Schmit KM , Guy GP Jr . Chronic pain among adults — United States, 2019–2021. MMWR Morb Mortal Wkly Rep. 2023;72:379‐385. doi:10.15585/mmwr.mm7215a1 PMC10121254 37053114

[2] Yong RJ , Mullins PM , Bhattacharyya N . Prevalence of chronic pain among adults in the United States. Pain. 2022;163:e328‐e332. doi:10.1097/j.pain.0000000000002291 33990113

[3] Dahlhamer J , Lucas J , Zelaya C , et al. Prevalence of chronic pain and high‐impact chronic pain among adults — United States, 2016. MMWR Morb Mortal Wkly Rep. 2018;67(36):1001‐1006. doi:10.15585/mmwr.mm6736a2 PMC6146950 30212442

[4] National Pain Strategy: A Comprehensive Population Health‐Level Strategy for Pain. Interagency Pain Research Coordinating Committee. Accessed April 26, 2024. https://www.iprcc.nih.gov/node/5/national-pain-strategy-report

[5] Cohen SP , Vase L , Hooten WM . Chronic pain: an update on burden, best practices, and new advances. Lancet. 2021;397(10289):2082‐2097. doi:10.1016/S0140-6736(21)00393-7 34062143

[6] Gatchel RJ , McGeary DD , McGeary CA , Lippe B . Interdisciplinary chronic pain management: past, present, and future. Am Psychol. 2014;69(2):119‐130. doi:10.1037/a0035514 24547798

[7] Elbers S , Wittink H , Konings S , et al. Longitudinal outcome evaluations of interdisciplinary multimodal pain treatment programmes for patients with chronic primary musculoskeletal pain: a systematic review and meta‐analysis. Eur J Pain. 2022;26(2):310‐335. doi:10.1002/ejp.1875 PMC9297911 34624159

[8] Di Lernia D , Serino S , Riva G . Pain in the body. Altered interoception in chronic pain conditions: a systematic review. Neurosci Biobehav Rev. 2016;71:328‐341. doi:10.1016/j.neubiorev.2016.09.015 27654341

[9] Horsburgh A , Summers SJ , Lewis A , Keegan RJ , Flood A . The relationship between pain and interoception: a systematic review and meta‐analysis. J Pain. 2024;25(7):104476. doi:10.1016/j.jpain.2024.01.341 38244898

[10] Garfinkel SN , Critchley HD . Interoception, emotion and brain: new insights link internal physiology to social behavior. Commentary on: “anterior insular cortex mediates bodily sensibility and social anxiety” by Terasawa et al. (2012). Scan. 2013;8:231‐234. doi:10.1093/scan/nss140 PMC3594730 23482658

[11] Mehling WE . Differentiating attention styles and regulatory aspects of self‐reported interoceptive sensibility. Philos Trans R Soc Lond Ser B Biol Sci. 2016;371:20160013. doi:10.1098/rstb.2016.0013 PMC5062101 28080970

[12] Gnall KE , Sinnott SM , Laumann LE , Park CL , David A , Emrich M . Changes in interoception in mind‐body therapies for chronic pain: a systematic review and meta‐analysis. Int J Behav Med. 2024;31:833‐847. doi:10.1007/s12529-023-10249-z 38169051

[13] Mehling WE , Acree M , Stewart A , Silas J , Jones A . The multidimensional assessment of interoceptive awareness, version 2 (MAIA‐2). PLoS One. 2018;13(12):1‐12. doi:10.1371/journal.pone.0208034 PMC6279042 30513087

[14] Mehling WE , Price C , Daubenmier J , Acree M , Bartmess E , Stewart A . The multidimensional assessment of interoceptive awareness (MAIA). PLoS One. 2012;7(11):e48230. doi:10.1371/journal.pone.0048230 PMC3486814 23133619

[15] Roberts RL , Ledermann K , Garland EL . Mindfulness‐oriented recovery enhancement improves negative emotion regulation among opioid‐treated chronic pain patients by increasing interoceptive awareness. J Psychosom Res. 2022;152:152. doi:10.1016/j.jpsychores.2021.110677 34801814

[16] Berry MP , Lutz J , Schuman‐Olivier Z , et al. Brief self‐compassion training alters neural responses to evoked pain for chronic low back pain: a pilot study. Pain Med. 2020;21(10):2172‐2185. doi:10.1093/pm/pnaa178 PMC7593799 32783054

[17] Ahmadi H , Adib H , Selk‐Ghaffari M , et al. Comparison of the effects of the Feldenkrais method versus core stability exercise in the management of chronic low back pain: a randomised control trial. Clin Rehabil. 2020;34(12):1449‐1457. doi:10.1177/0269215520947069 32723088

[18] Patient‐Reported Outcomes Measurement Information System (PROMIS). National Institute on Aging. https://www.nia.nih.gov/research/resource/patient‐reported‐outcomes‐measurement‐information‐system‐promis

[19] Law M , Baptiste S , Mccoll M , Opzoomer A , Polatajko H , Pollock N . The Canadian occupational performance measure: an outcome measure for occupational therapy. Can J Occup Ther. 1990;57(2):82‐87. doi:10.1177/000841749005700207 10104738

[20] Stratford P , Gill C , Westaway M , Binkley J . Assessing disability and change on individual patients: a report of a patient specific measure. Physiother Can. 1995;47(4):258‐263. doi:10.3138/ptc.47.4.258

[21] Bornemann B , Herbert BM , Mehling WE , Singer T . Differential changes in self‐reported aspects of interoceptive awareness through 3 months of contemplative training. Front Psychol. 2014;5:1‐13. doi:10.3389/fpsyg.2014.01504 PMC4284997 25610410

[22] Osypiuk K , Ligibel J , Giobbie‐Hurder A , et al. Qigong mind‐body exercise as a biopsychosocial therapy for persistent post‐surgical pain in breast cancer: a pilot study. Integr Cancer Ther. 2020;19:1‐12. doi:10.1177/1534735419893766 PMC7050958 32009481

[23] Dunlap WP , Cortina JM , Vaslow JB , Burke MJ . Meta‐analysis of experiments with matched groups or repeated measures designs. Psychol Methods. 1996;1(2):170‐177. doi:10.1037/1082-989X.1.2.170

[24] Cohen J . Statistical Power Analysis for the Behavioral Sciences. 2nd ed. Lawrence Earlbaum Associates; 1988.

[25] Maughan E , Lewis J . Outcome measures in chronic low back pain. Eur Spine J. 2010;19:1484‐1494. doi:10.1007/s00586-010-1353-6 PMC2989277 20397032

[26] Terwee CB , Peipert JD , Chapman R , et al. Minimal important change (MIC): a conceptual clarification and systematic review of MIC estimates of PROMIS measures. Qual Life Res. 2021;30(10):2729‐2754. doi:10.1007/s11136-021-02925-y PMC8481206 34247326

[27] Lauche R , Stumpe C , Fehr J , et al. The effects of tai chi and neck exercises in the treatment of chronic nonspecific neck pain: a randomized controlled trial. J Pain. 2016;17(9):1013‐1027. doi:10.1016/j.jpain.2016.06.004 27345663

[28] de Jong M , Lazar SW , Hug K , et al. Effects of mindfulness‐based cognitive therapy on body awareness in patients with chronic pain and comorbid depression. Front Psychol. 2016;7:13. doi:10.3389/fpsyg.2016.00967 PMC4927571 27445929

[29] Edwards RR , Dworkin RH , Sullivan MD , Turk DC , Wasan AD . The role of psychosocial processes in the development and maintenance of chronic pain. J Pain. 2016;17(9):T70‐T92. doi:10.1016/j.jpain.2016.01.001 PMC5012303 27586832

[30] Gagnon CM , Yuen M , Palmer K . An exploration of physical therapy outcomes and psychometric properties of the patient‐specific functional scale after an interdisciplinary pain management program. Clin J Pain. 2023;39(12):663‐671. doi:10.1097/AJP.0000000000001159 37712294

[31] Carswell A , McColl MA , Baptiste S , Law M , Polatajko H , Pollock N . The Canadian occupational performance measure: a research and clinical literature review. Can J Occup Ther. 2004;71(4):210‐222. doi:10.1177/000841740407100406 15586853

[32] Carpenter L , Baker GA , Tyldesley B . The use of the Canadian Occupational Performance Measure as an outcome of a pain management program. Can J Occup Ther. 2001;68(1):16‐22. doi:10.1177/000841740106800102 11233684

[33] Dedding C , Cardol M , Eyssen ICJM , Dekker J , Beelen A . Validity of the Canadian occupational performance measure: a client‐centered outcome measurement. Clin Rehabil. 2004;18(6):660‐667. doi:10.1191/0269215504cr746oa 15473118

[34] Schmitz M , Back SN , Seitz KI , et al. The impact of traumatic childhood experiences on interoception: disregarding one's own body. Borderline Personal Disord Emot Dysregul. 2023;10(1):5. doi:10.1186/s40479-023-00212-5 PMC9930318 36788573

[35] Freedman A , Hu H , Liu ITHC , Stewart AL , Adler S , Mehling WE . Similarities and differences in interoceptive bodily awareness between US‐American and Japanese cultures: a focus‐group study in bicultural Japanese‐Americans. Cult Med Psychiatry. 2021;45(2):234‐267. doi:10.1007/s11013-020-09684-4 32740780

[36] Murphy J , Viding E , Bird G . Does atypical interoception following physical change contribute to sex differences in mental illness? Psychol Rev. 2019;126(5):787‐789. doi:10.1037/rev0000158 31380676

[37] Garfinkel SN , Tiley C , O'Keeffe S , Harrison NA , Seth AK , Critchley HD . Discrepancies between dimensions of interoception in autism: implications for emotion and anxiety. Biol Psychol. 2016;114:117‐126. doi:10.1016/j.biopsycho.2015.12.003 26724504

[38] Bruton MA , Levy L , Rai NK , Colgan DD , Johnstone M . Diminished interoceptive accuracy in attention‐deficit/hyperactivity disorder: a systematic review. Psychophysiology. 2025;62(2):e14750. doi:10.1111/psyp.14750 PMC11842156 39905593

[39] Schrepf A , Williams DA , Gallop R , et al. Sensory sensitivity and symptom severity represent unique dimensions of chronic pain: a MAPP research network study. Pain. 2018;159(10):2002‐2011. doi:10.1097/j.pain.0000000000001299 PMC6705610 29863527

[40] Schrepf A , Hellman KM , Bohnert AM , Williams DA , Tu FF . Generalized sensory sensitivity is associated with comorbid pain symptoms: a replication study in women with dysmenorrhea. Pain. 2023;164(1):142‐148. doi:10.1097/j.pain.0000000000002676 PMC9704354 35543649

[41] Wang D , Frey‐Law LA . Multisensory sensitivity differentiates between multiple chronic pain conditions and pain‐free individuals. Pain. 2023;164(2):e91. doi:10.1097/j.pain.0000000000002696 PMC11075969 35588150

