# Supplementary material for: Improvements in interoceptive sensibility, pain and functional clinical outcome measures after participation in an interdisciplinary pain management program
Source: PM R. 2026 Mar 16;18(10):1122–32. doi: 10.1002/pmrj.70110 (PMC13686118; doi:10.1002/pmrj.70110)
Supplement: Supplementary file 1 [file PMRJ-18-1122-s001.docx]

**Supplementary Materials**

**Measures: Scoring and Psychometrics**

**The Multidimensional Assessment of Interoceptive Awareness, Version 2 (MAIA-2)**

The MAIA-2 is a 37-item questionnaire measuring eight domains of interoceptive sensibility, including: Noticing, Not Distracting, Not Worrying, Attention Regulation, Emotional Awareness, Self-Regulation, Body Listing and Trusting^1^. Participants rate how much each statement applies to them generally in daily life from 0 (Never) to 5 (Always). Total and subscale scores are calculated from the mean of item responses. Higher scores indicate greater interoceptive sensibility. All MAIA-2 subscales demonstrate acceptable internal consistency in healthy individuals (α’s = 0.64-0.83)^1^ and were similarly acceptable in this sample (α’s = 0.68-0.91). The original MAIA subscales have demonstrated high test-retest reliability (r’s = 0.66-0.79) and sensitivity to change following contemplative training in healthy individuals^2^. In this sample, the full MAIA-2 demonstrated good internal consistency (α = 0.89), suggesting that an overall mean appropriately represented overall interoceptive sensibility. Subscale descriptions and Cronbach’s alphas are listed in Supplementary Table 1.

**Patient Reported Outcomes Measurement Information System (PROMIS) – Pain Intensity and Pain Interference Forms**

The PROMIS Pain Intensity and Pain Interference forms are part of the NIH toolbox of patient-reported outcomes^3^. The Pain Intensity measure is a 3-item questionnaire, providing a quantitative assessment of pain intensity over the past 7 days at its worst, average, and “right now.” The Pain Interference Short Form 8a is an 8-item questionnaire providing a global rating of how much pain interferes with daily life, including activities of daily living, social activities, and leisure. Individual items are rated on a 5-point scale from 1 (No pain/No interference at all, respectively) to 5 (Very severe pain/Pain interferes very much, respectively). Individual scores are summed and correspond to T-Scores (mean = 50, sd = 10), with higher scores indicating greater levels of pain intensity and interference. These measures demonstrate good-excellent reliability and validity^4–6^. In this sample, the pain measures demonstrated good-excellent internal consistency (Intensity α = 0.71, Interference α = 0.92).

**Canadian Occupational Performance Measure (COPM)**

The COPM is a semi-structured interview designed to assess an individual’s perceived level of performance and satisfaction with up to five valued occupations in the context of injury or illness^7^. Both Performance and Satisfaction are rated from 1-10 for each activity, where a score of 1 represents poor performance or low satisfaction, respectively. Ratings are summed and divided by the number of responses to yield a total score. Higher scores indicate greater perceived performance and satisfaction with patient-valued functional goals. The COPM demonstrates excellent test-retest reliability (ICCs: 0.88-0.93)^8,9^ and good responsiveness to change with chronic pain patients in outpatient settings^10,11^. In this sample, the COPM-Performance scale demonstrated unexpectedly low internal consistency at evaluation but improved substantially at discharge (α’s = 0.502 and 0.859, respectively). The COPM-Satisfaction scale demonstrated acceptable internal consistency at both evaluation and discharge (α’s = 0.708 and 0.869, respectively).

**Patient-Specific Functional Scale (PSFS)**

The PSFS measures a patient’s subjective report of functional impairments due to an injury or illness with up to 3 patient-specific functional goals. Functional impairment for each activity is rated on a scale of 0 (Unable to perform) to 10 (Able to perform at prior level/no difficulty). Ratings are summed and divided by the number of responses to yield a total score. Lower scores indicate greater functional limitation. The PSFS demonstrates excellent test-retest reliability^12–14^ and was originally validated against the Roland Morris Questionnaire^12^ and the physical function, bodily pain, and social function dimensions of the 36-Item Short Form Health survey^14^. In this sample, the PSFS demonstrated unexpectedly low internal consistency at evaluation but improved substantially at discharge (α’s = 0.525 and 0.811, respectively).

**Supplemental Tables**

**Supplemental Table 1:** MAIA-2 Subscale Descriptions and Cronbach’s Alphas

| Scale Descriptions^1^ | Alphas |
| --- | --- |
| **Noticing:** Awareness of uncomfortable, comfortable, and neutral sensations. | 0.68 |
| **Not Distracting:** Tendency not to ignore or distract oneself from sensations of pain or discomfort. | 0.82 |
| **Not Worrying:** Tendency not to worry or experience emotional distress with sensations of pain or discomfort. | 0.76 |
| **Attention Regulation:** Ability to sustain and control attention to body sensations. | 0.88 |
| **Emotional Awareness:** Awareness of the connection between body sensations and emotional states. | 0.76 |
| **Self-Regulation**: Ability to regulate distress by attention to body sensations. | 0.81 |
| **Body Listening:** Active listening to the body for insight. | 0.79 |
| **Trusting:** Experience of one’s body as safe and trustworthy. | 0.91 |
| **Total MAIA-2:** Mean of full scale. | 0.89 |

*Note:* Cronbach alphas for MAIA-2 subscales in heterogeneous chronic pain. Scale descriptions reproduced from the original MAIA-2 article^1^. Abbreviations: MAIA-2 (Multidimensional Assessment of Interoceptive Awareness, Version 2).

**Supplemental Table 2:** Baseline Scores for Interoceptive Sensibility, Pain and Functional Measures

| Measure | All Participants | Completers | Non-Completers | *p* |
| --- | --- | --- | --- | --- |
| N | 63 | 53 | 10 |  |
| MAIA-2, Overall  Noticing  Not Distracting  Not Worrying  Attention Regulation  Emotional Awareness  Self-Regulation  Body Listening  Trusting | 2.61 (0.61)  3.37 (0.97)  1.74 (0.88)  2.39 (1.03)  2.80 (1.02)  3.28 (0.95)  2.45 (1.09)  2.56 (1.27)  2.44 (1.49) | 2.57 (0.60)  3.35 (1.01)  1.75 (0.89)  2.26 (0.97)  2.69 (1.00)  3.28 (0.97)  2.41 (1.01)  2.51 (1.22)  2.35 (1.47) | 2.94 (0.58)  3.47 (0.80)  1.69 (0.84)  3.18 (1.07)  3.43 (0.97)  3.29 (0.83)  2.72 (1.56)  2.89 (1.55)  2.96 (1.58) | 0.08  0.73  0.84  0.01*  0.04*  0.97  0.57  0.41  0.26 |
| Pain  Intensity  Interference | 56.12 (4.74)  66.43 (5.73) | 56.51 (4.85)  66.59 (5.46) | 54.12 (3.58)  65.48 (7.48) | 0.16  0.60 |
| Functional Outcomes  COPM-P  COPM-S  PSFS | 3.43 (1.01)  2.59 (1.24)  3.15 (1.15) | 3.51 (1.01)  2.60 (1.31)  3.13 (1.20) | 2.85 (0.87)  2.46 (0.72)  3.22 (0.94) | 0.08  0.68  0.83 |

*Note:* Means (standard deviations) for interoceptive sensibility, pain intensity and interference, and functional clinical outcome measures at program start. P-values for independent sample t-test between program completers and non-completers. **p* < .05, Abbreviations: MAIA-2 (Multidimensional Assessment of Interoceptive Awareness, Version 2); COPM-P/S (Canadian Occupational Performance Measure – Performance/Satisfaction); PSFS (Patient Specific Functional Scale), IPMP (Interdisciplinary Pain Management Program).

**Supplemental Table 3:** Exploratory Partial Correlations Between Interoceptive Sensibility, Pain, and Functional Measures at Baseline, Controlled for Age

|  | MAIA-2 | Intensity | Interference | COPM-P | COPM-S |
| --- | --- | --- | --- | --- | --- |
| Pain (N=53)  Intensity  Interference | 0.151  0.058 | -  0.635*** | -  - | -  - | -  - |
| Functional Outcomes  COPM-P (N=53)  COPM-S (N=53)  PSFS (N=49) | 0.282*  0.297*  0.268 | -0.063  -0.110  -0.299* | -0.242  -0.395**  -0.237 | -  0.664***  0.215 | -  -  0.250 |

*Note:* Pearson’s correlation coefficients, controlled for age. **p* < .05, ***p* < .01, ****p* < .001. Abbreviations: MAIA-2 (Multidimensional Assessment of Interoceptive Awareness, Version 2); COPM-P/S (Canadian Occupational Performance Measure – Performance/Satisfaction); PSFS (Patient Specific Functional Scale).

**Supplemental Table 4:** Exploratory Partial Correlations of MAIA-2 Subscales with Pain and Clinical Measures at Baseline, Controlled for Age

| MAIA-2 Subscale | Intensity (N=53) | Interference  (N=53) | COPM-P  (N=53) | COPM-S  (N=53) | PSFS  (N=49) |
| --- | --- | --- | --- | --- | --- |
| Noticing | 0.**429*** | **0.378**** | 0.053 | -0.034 | 0.047 |
| Not Distracting | -0.216 | -0.174 | -0.105 | 0.017 | -0.130 |
| Not Worrying | -0.002 | -0.198 | 0.107 | 0.170 | 0.112 |
| Attention Regulation | 0.145 | 0.158 | 0.218 | 0.205 | 0.191 |
| Emotional Awareness | 0.176 | 0.143 | **0.332*** | 0.247 | 0.226 |
| Self-Regulation | 0.032 | -0.089 | 0.227 | 0.272 | 0.**358*** |
| Body Listening | **0.353*** | 0.**274*** | 0.255 | 0.221 | 0.204 |
| Trusting | -0.103 | -0.168 | 0.265 | **0.302*** | **0.314*** |

*Note:* Pearson correlation coefficients, controlled for age. **Bold text** indicates significant correlations. **p* < .05, ***p*< .01. Abbreviations: MAIA-2 (Multidimensional Assessment of Interoceptive Awareness, Version 2); COPM-P/S (Canadian Occupational Performance Measure – Performance/ Satisfaction); PSFS (Patient Specific Functional Scale).

**Supplemental References**

1.	Mehling WE, Acree M, Stewart A, Silas J, Jones A. The multidimensional assessment of interoceptive awareness, version 2 (MAIA-2). *PLoS ONE*. 2018;13(12):1-12. doi:10.1371/journal.pone.0208034

2.	Bornemann B, Herbert BM, Mehling WE, Singer T. Differential changes in self-reported aspects of interoceptive awareness through 3 months of contemplative training. *Front Psychol*. 2014;5(OCT):1-13. doi:10.3389/fpsyg.2014.01504

3.	Patient-Reported Outcomes Measurement Information System (PROMIS). National Institute on Aging. https://www.nia.nih.gov/research/resource/patient-reported-outcomes-measurement-information-system-promis

4.	Amtmann D, Cook KF, Jensen MP, et al. Development of a PROMIS item bank to measure pain interference. *Pain*. 2010;150(1):173-182. doi:10.1016/j.pain.2010.04.025

5.	Cella D, Riley W, Stone A, et al. The patient-reported outcomes measurement information system (PROMIS) developed and tested its first wave of adult self-reported health outcome item banks: 2005–2008. *J Clin Epidemiol*. 2010;63(11):1179-1194. doi:10.1016/j.jclinepi.2010.04.011

6.	Purvis TE, Neuman BJ, Riley LHI, Skolasky RL. Discriminant ability, concurrent validity, and responsiveness of PROMIS health domains among patients with lumbar degenerative disease undergoing decompression with or without arthrodesis. *Spine*. 2018;43(21):1512. doi:10.1097/BRS.0000000000002661

7.	Law M, Baptiste S, Mccoll M, Opzoomer A, Polatajko H, Pollock N. The Canadian Occupational Performance Measure: An Outcome Measure for Occupational Therapy. *Can J Occup Ther*. 1990;57(2):82-87. doi:10.1177/000841749005700207

8.	Cup EHC, Scholte op Reimer WJM, Thijssen MCE, van Kuyk-Minis M a. H. Reliability and validity of the Canadian occupational performance measure in stroke patients. *Clin Rehabil*. 2003;17(4):402-409. doi:10.1191/0269215503cr635oa

9.	Kjeken I, Dagfinrud H, Uhlig T, Mowinckel P, Kvien TK, Finset A. Reliability of the Canadian occupational performance measure in patients with ankylosing spondylitis. *J Rheumatol*. 2005;32(8):1503-1509.

10.	Carpenter L, Baker GA, Tyldesley B. The use of the Canadian Occupational Performance Measure as an outcome of a Pain Management Program. *Can J Occup Ther*. 2001;68(1):16-22. doi:10.1177/000841740106800102

11.	Nieuwenhuizen MG, de Groot S, Janssen TWJ, van der Maas LCC, Beckerman H. Canadian occupational performance measure performance scale: validity and responsiveness in chronic pain. *J Rehabil Res Dev*. 2014;51(5):727-746. doi:10.1682/JRRD.2012.12.0221

12.	Stratford P, Gill C, Westaway M, Binkley J. Assessing disability and change on individual patients: a report of a patient specific measure. *Physiother Can*. 1995;47(4):258-263. doi:10.3138/ptc.47.4.258

13.	Horn KK, Jennings S, Richardson G, van Vliet D, Hefford C, Abbott JH. The patient-specific functional scale: psychometrics, clinimetrics, and application as a clinical outcome measure. *J Orthop Sports Phys Ther*. 2012;42(1):30-42. doi:10.2519/jospt.2012.3727

14.	Chatman AB, Hyams SP, Neel JM, et al. The patient-specific functional scale: measurement properties in patients with knee dysfunction. *Phys Ther*. 1997;77(8):820-829. doi:10.1093/ptj/77.8.820
